# Feasibility of a Mobile Health App for Routine Outcome Monitoring and Feedback in Mutual Support Groups Coordinated by SMART Recovery Australia: Protocol for a Pilot Study

**DOI:** 10.2196/15113

**Published:** 2020-07-09

**Authors:** Peter J Kelly, Alison K Beck, Amanda L Baker, Frank P Deane, Leanne Hides, Victoria Manning, Anthony Shakeshaft, Briony Larance, Joanne Neale, John Kelly, Christopher Oldmeadow, Andrew Searles, Carla Treloar, Rebecca M Gray, Angela Argent, Ryan McGlaughlin

**Affiliations:** 1 Faculty of Social Sciences School of Psychology University of Wollongong Wollongong Australia; 2 Illawarra Health and Medical Research Institute Wollongong Australia; 3 School of Medicine and Public Health University of Newcastle Callaghan Australia; 4 Centre for Youth Substance Abuse Research, Lives Lived Well Group School of Psychology University of Queensland Brisbane St Lucia Australia; 5 Faculty of Medicine, Nursing and Health Sciences Eastern Health Clinical School Monash University Melbourne Australia; 6 Faculty of Arts and Social Sciences Centre for Social Research in Health University of New South Wales Sydney Australia; 7 Institute of Psychiatry, Psychology and Neuroscience King’s College London London United Kingdom; 8 Centre for Addiction Medicine Harvard Medical School Harvard University Boston, MA United States; 9 Clinical Research Design, IT and Statistical Support Unit Hunter Medical Research Institute New Lambton Australia; 10 Health Research Economics Unit Hunter Medical Research Institute New Lambton Australia; 11 SMART Recovery Australia Sydney Australia

**Keywords:** SMART Recovery, mutual support group, mutual aid, routine outcome monitoring, treatment progress feedback, mHealth, addiction, mobile phone

## Abstract

**Background:**

Despite the importance and popularity of mutual support groups, there have been no systematic attempts to implement and evaluate routine outcome monitoring (ROM) in these settings. Unlike other mutual support groups for addiction, trained facilitators lead all Self-Management and Recovery Training (SMART Recovery) groups, thereby providing an opportunity to implement ROM as a routine component of SMART Recovery groups.

**Objective:**

This study protocol aims to describe a stage 1 pilot study designed to explore the feasibility and acceptability of a novel, purpose-built mobile health (mHealth) ROM and feedback app (Smart Track) in SMART Recovery groups coordinated by SMART Recovery Australia (SRAU) The secondary objectives are to describe Smart Track usage patterns, explore psychometric properties of the ROM items (ie, internal reliability and convergent and divergent validity), and provide preliminary evidence for participant reported outcomes (such as alcohol and other drug use, self-reported recovery, and mental health).

**Methods:**

Participants (n=100) from the SMART Recovery groups across New South Wales, Australia, will be recruited to a nonrandomized, prospective, single-arm trial of the Smart Track app. There are 4 modes of data collection: (1) ROM data collected from group participants via the Smart Track app, (2) data analytics summarizing user interactions with Smart Track, (3) quantitative interview and survey data of group participants (baseline, 2-week follow-up, and 2-month follow-up), and (4) qualitative interviews with group participants (n=20) and facilitators (n=10). Feasibility and acceptability (primary objectives) will be analyzed using descriptive statistics, a cost analysis, and a qualitative evaluation.

**Results:**

At the time of submission, 13 sites (25 groups per week) had agreed to be involved. Funding was awarded on August 14, 2017, and ethics approval was granted on April 26, 2018 (HREC/18/WGONG/34; 2018/099). Enrollment is due to commence in July 2019. Data collection is due to be finalized in October 2019.

**Conclusions:**

To the best of our knowledge, this study is the first to use ROM and tailored feedback within a mutual support group setting for addictive behaviors. Our study design will provide an opportunity to identify the acceptability of a novel mHealth ROM and feedback app within this setting and provide detailed information on what factors promote or hinder ROM usage within this context. This project aims to offer a new tool, should Smart Track prove feasible and acceptable, that service providers, policy makers, and researchers could use in the future to understand the impact of SMART Recovery groups.

**Trial Registration:**

Australian New Zealand Clinical Trials Registry (ANZCTR): ACTRN12619000686101; https://anzctr.org.au/Trial/Registration/TrialReview.aspx?id=377336.

**International Registered Report Identifier (IRRID):**

PRR1-10.2196/15113

## Introduction

### Background

Using standardized outcome measures to regularly monitor client progress in alcohol and other drug (AOD) settings is an important mechanism for monitoring the effectiveness of service provision [1-3]. Routine outcome monitoring (ROM) provides clinicians with timely feedback about client progress and allows clinicians to tailor treatment to the individual needs of clients and guide treatment decisions [4]. This may be of particular importance when a client is *not on track* (ie, not improving in line with clinical norms [4]). ROM has been specifically recommended for use in AOD treatment settings as the provision of tailored feedback to clients has been found to improve treatment outcomes across a range of AOD treatment settings (eg, acute, community, veterans) [3,5,6]. Evidence also supports clinician use of ROM and tailored feedback to enhance outcomes and/or prevent further deterioration for those clients identified as *not on track* early in addiction and/or mental health treatment [7,8].

Despite the importance of ROM and tailored feedback, ongoing variability in the implementation, sustainability, and use of ROM data has been noted [9]. The time associated with the completion, scoring, interpretation, and feedback of outcome assessments represents a key barrier to systematic implementation [6,9]. Using a digital platform to administer ROM and feedback may help address these concerns. Mobile health (mHealth) [10] apps can provide quick, easy, interactive, and engaging platforms for tracking and accessing information about health and health-related behaviors [11]. Evidence from the United Kingdom suggests that almost 60% of individuals who access AOD treatment own a smartphone [12]. This figure is likely higher in Australia as it is the leading global adopter of smartphones (88% ownership [13]). Given the ubiquity of smartphones, smartphone apps have the added benefit of engaging individuals in real time, in their natural environment, and offering moment-to-moment support as needed [14]. Moreover, a key benefit highlighted in a recent systematic review of mHealth apps is their ability to provide timely, individualized feedback [15]. Accordingly, an opportunity exists to utilize mHealth to enhance engagement, streamline administration, and put the client at the center of the ROM and feedback process.

To date, much of the literature on ROM and feedback has focused on the provision and use of feedback by clinicians [16,17]. Improving client involvement in the feedback process represents an important clinical and research priority [18]. It is not only consistent with the principles of recovery-oriented service provision and strengths-based care [19] but is also therapeutically useful. Within mental health settings, the benefits of providing clients with outcome feedback include improved client insight; enhanced knowledge, skill, and confidence to effectively self-manage their condition(s); and greater satisfaction, engagement, and involvement in treatment [16]. Evidence from related approaches (eg, *therapeutic assessment* [20]) has also shown that providing clients with assessment feedback during psychotherapy promotes client self-verification, self-discovery, and self-enhancement [18]. Moreover, delivering feedback directly to the client seems to further enhance the positive impact of ROM on treatment outcome(s), particularly among group-based treatment settings [6]. Therefore, to meet this important need of putting the client at the center of the ROM and feedback process, during the first phase of this study, we developed a purpose-built mHealth app.

### Mutual Support Groups

*Mutual support* refers to the reciprocal provision of social, emotional, and informational support by group members undergoing recovery from addiction [21]. Mutual support groups are widely available [22], commonly accessed [23], and play an extremely important role in the treatment of AOD use disorders [2,24]. Two approaches recommended by clinical guidelines include 12-step models (eg, Alcoholics Anonymous) and Self-Management and Recovery Training (*SMART Recovery* [2,24]). Although the 12-step models are traditionally the most well-known and accessed models for mutual support [22], other approaches (eg, SMART Recovery) are gaining momentum. For example, SMART Recovery Australia has seen an almost 40% increase in groups over the last 4 years, with over 300 groups currently running nationwide [25]. Although accumulating evidence points to the benefit of participating in mutual support groups [26,27], much of the research is derived from the 12-step models. In light of the growth of SMART Recovery groups, expanding the evidence base beyond the 12-step models is a priority. A major limitation in developing a strong evidence base is the lack of outcome data evaluating service delivery. Accordingly, the purpose-built mHealth app developed during the first phase of the study provides a mechanism for not only improving service provision but also providing unique insights into the outcome(s) demonstrated by SMART Recovery participants.

Although many AOD services provided by public health and nongovernment organizations are contracted to monitor client outcomes routinely [28], we are unaware of any research describing the use of ROM in mutual support groups for addictive behaviors. As a trained facilitator leads all SMART Recovery groups, a unique opportunity exists to work with SMART Recovery facilitators to embed ROM and tailored feedback as a standard component of the groups. Investigating the use of ROM and feedback within a mutual support setting helps address the need for improved participant involvement in the assessment and feedback process [18], in addition to building a platform for improving the evidence base for SMART Recovery, a clinical and research priority [27].

### This Study

In this paper, we detail the study protocol for a nonrandomized, prospective, single-arm pilot study, with a concurrent cost evaluation and nested qualitative evaluation designed to explore the feasibility and acceptability of a novel mHealth ROM and feedback app (*Smart Track*) in SMART Recovery groups coordinated by SRAU. The secondary objectives are to describe Smart Track usage patterns, psychometric properties of the ROM items, and participant-reported outcomes.

## Methods

### Approval, Registration, and Reporting

This study was approved by the University of Wollongong and Illawarra Shoalhaven Local Health District (ISLHD) Health and Medical Human Research Ethics Committee (HREC; 2018/099; HREC/18/WGONG/34). The trial has been registered with the Australian New Zealand Clinical Trials Registry (ACTRN12619000686101). Any amendments will be submitted to the ISLHD HREC before implementation, as per HREC guidelines. Any important protocol modifications will be reported in the outcomes paper. To enhance the quality, completeness, and transparency of the proposed study, this study protocol follows the Standard Protocol Items: Recommendations for Interventional Trials [29] and the Consolidated Standards of Reporting Trials-eHealth checklist [30] (Multimedia Appendix 1).

### Participants

#### Eligibility

Participants must be at least 18 years of age, currently participating in one or more SMART Recovery groups located within New South Wales (NSW), have a current email address or be willing to obtain an email address, and be able to comprehend English at a level sufficient to complete study requirements.

Participants will be eligible irrespective of their self-reported computer and/or smartphone literacy. No restrictions will be placed on concomitant care, including the frequency or duration of SMART Recovery group participation or participation in other forms of AOD treatment. Participants will only be excluded if they are unable or unwilling to provide informed consent. Exclusion criteria were kept to a minimum to ensure that the study sample is representative of people attending SMART Recovery groups.

#### Smartphone Ownership

Although we expect that most participants will own a smartphone [12,13], potential participants do not need to own a smartphone to participate. The research team will provide tablets to study sites (locations where regular SMART Recovery groups are held) so that participants can use the tablet before and/or after attending a group to complete the ROM questions and receive feedback.

### Study Setting

Potential participants will be sourced from the SMART Recovery groups held in NSW, Australia. A full list of study sites will be reported in the outcomes paper. SMART Recovery groups are held in the community as well as in inpatient, outpatient, and clinical health organizations, including private, public, and not-for-profit mental health, AOD, and general health services. Online SMART Recovery groups are also available. A detailed description of the SMART Recovery groups has been provided elsewhere [31]. Briefly, SMART Recovery focuses on self-empowerment and utilizes evidence-based techniques (eg, cognitive behavioral therapy and motivational interviewing) [32]. To ensure that our sample adequately reflects SMART Recovery participants, study sites with established SMART Recovery groups were selected to reflect a range of geographical locations and service providers. At the time of manuscript submission, 147 groups were conducted throughout the NSW.

### Enrollment

Group facilitators will use a script to introduce the study to potential participants and invite expressions of interest. The following strategies will be adopted to maximize adequate enrollment. Group facilitators will be asked to check in with participants regarding the completion and/or return of expression of interest forms (across a maximum of 3 meetings). A member of the research team will also visit SMART Recovery groups throughout the recruitment period to directly provide group members with information about the study and collect the expression of interest forms. Depending on accrual, the study may also be advertised (eg, online, local media, flyers/pamphlets, and study website) to extend participant recruitment beyond this study’s sites.

### Informed Consent

A member of the research team (AKB) will collect informed consent from all study participants (written or verbal according to the participant’s preference). A copy of consent (audio recording and/or signed consent form) will be retained for all study participants and securely stored according to the HREC–approved methods.

### Overview of the mHealth Routine Outcome Monitoring and Feedback App (Smart Track)

The Smart Track app was designed for participants attending SMART Recovery groups. ROM items are intended for weekly completion. However, it is up to the individual to decide whether, when, and how they engage with Smart Track. Given the low-risk, low-burden nature of this study, there are no contingencies for discontinuing access to the Smart Track app. Owing to app store regulations, Smart Track will not be restricted to study participants. It will be freely available for download through Android and iPhone Operating System stores (only data from those who provide consent will be included in the study).

Publications detailing the methods and findings from the qualitative work [33] and app development process (including theoretical foundations) will be reported separately. To provide context, a brief summary is presented here. The Smart Track app was developed during the first phase of this study using participatory design workshops and an iterative development process informed by the (1) consideration of existing ROM tools, (2) qualitative feedback [33] and usability testing sessions with SMART Recovery participants and facilitators, (3) clinical and research expertise from the members of the expert advisory and steering committees, and (4) technological and creative expertise of the development team employed to work on this study (GHO, Sydney) [34].

The functionality of Smart Track was initially tested with 3 members of the research team. Several bugs were identified and fixed before the amended beta version was released to a convenience sample (n=40) for further testing. This convenience sample of beta testers included members of the expert advisory committee, steering committee, SMART Recovery board members, and SMART Recovery facilitators. Further refinements were made in line with the feedback received (bug fixes, minor amendments to functionality, and content).

#### Smart Track Routine Outcome Monitoring Domains and Items

Consistent with clinical guidelines [3,35] and informed by recommendations arising from systematic reviews evaluating ROM in both mental health [18] and addiction [6] settings, we sought to create a tool that provided multidimensional assessment and feedback. Utilizing an iterative process, we generated a list of candidate outcomes (group attendance; goal setting and attainment; values; self-efficacy; quality of life; self-care; mental health; quantity, frequency, and impact of addictive behavior(s); social support; financial stability; optimism; and frequency, strength, and duration of urges). Corresponding assessment items and/or instruments were then identified to measure these domains. Where possible, validated free-to-access measurement instruments were selected from the published literature. Some changes to the structure, wording, and/or response format were required to improve the clarity and appropriateness of some items. The final item set included in the tool is detailed in Multimedia Appendix 2 [36-47] as a function of the target domain and assessment frequency.

#### Smart Track Tailored Feedback

The same iterative process was used to inform the content, format, and frequency of the Smart Track feedback. On the basis of the participants’ responses to individual ROM items and/or subscale scores, tailored visual cues (eg, colors and arrows) are used to provide a *snapshot* of progress within each domain (eg, current score range and/or direction of progress). Participants can also select one or more domains to receive further detailed feedback (written and visual).

The written feedback comprises encouraging statements, self-reflection questions, and/or self-management suggestions. As health messages are more effective when they are tailored to the individual [48] and tailored feedback is central to both popularity [49] and effectiveness [14] of mHealth apps for alcohol use, written feedback and visual cues are tailored. Visual feedback comprises a graph illustrating the participant’s progress over time (with the option of viewing data for the week, month, and year). Guided by the literature highlighting the utility of providing feedback according to the level of observed progress (eg, *on-track* or *off-track* [16]) and informed by previous *stop-light*–style ROM feedback systems (see the study by Kendrick et al [7] for a review), written feedback and visual cues are tailored according to three pathways, based on whether individual domain scores suggest (1) a *good* score range or improvement (green), (2) an *ok* score range or stability (yellow), or (3) a *less good* score range or deterioration (red). The chosen logic allows feedback content and visual cues to be tailored such that all progress is encouraged and reinforced, with a specific focus on (1) maintaining change (green), (2) highlighting additional change(s) that may be of benefit (yellow), or (3) troubleshooting difficulties and/or seeking support (red).

#### Additional Features of Smart Track

Inconsistent engagement (client and/or clinician) has long been identified as a challenge to both ROM and feedback [50] and, more broadly, the mHealth literature [51]. Therefore, in addition to the core ROM and feedback functionality, several additional features (Table 1) have been included. Aside from the hints, tips, and motivational statements (which automatically feature once per day that the app is used), it is up to the individual as to how frequently they access various features.

**Table 1:** Additional Smart Track features.

Feature	Description
Customizable support(s) and personal motivation(s)	Participants have the option of tailoring the app content by uploading key contact number(s), support services and/or personal motivation(s) for change (photo, audio, video, and/or written)
Resources	Information about self-management strategies (including SMART^a^ Recovery resources) and motivational stories from people with lived experience of addictive behavior(s)
Hints, tips, and motivational statements	A self-management tip, motivational statement, or inspirational quote will be included as pop-up content. These brief messages comprise direct and adapted quotes from the transcripts of the qualitative interviews
Journal	There is a free text box on each feedback page to allow participants to reflect on their progress and/or the tailored feedback provided
Interactive urge log	In addition to tracking the number, frequency, and strength of urges, when the participant reports an urge, this interactive tool prompts the participants to manage their urges, log triggers, and reflect on how to maintain and/or improve effective urge self-management

^a^SMART: Self-Management and Recovery Training.

#### Implementation Strategies

The app contains an in-built *walk through* to orient new users to the features of the app. We also intend to develop a brief tutorial to assist participants with app download and set up. Consistent with the recommendations for improving ROM uptake [9], SMART Recovery facilitators at each study site will assume the role of *local champions* of Smart Track. The research team will work with the facilitators to orient them to the app so that they are confident in responding to the participants’ questions about ROM completion and troubleshooting any difficulties that may arise. Participants and facilitators may also contact the research team directly for support. SMART Recovery facilitators will also prompt, encourage, and support study participants to regularly complete the ROM questions before and/or attending a SMART Recovery group.

In-app push notifications will also be used to prompt participants to complete the ROM items. In-app notifications (red marker) will also be used to highlight section(s) that require participants’ attention (eg, outstanding 7-day plan).

### Data Collection Procedures

**Figure 1:**
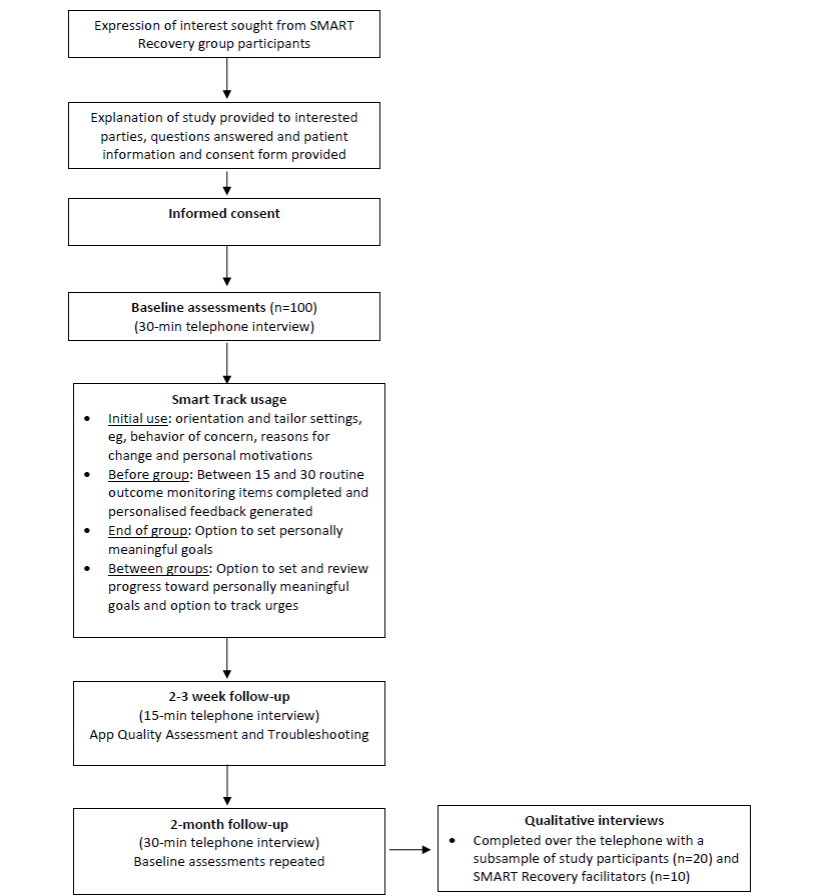
Participant flow chart. SMART: Self-Management and Recovery Training.

The participant timeline is outlined in Figure 1, and the corresponding schedule of participant assessments is summarized in Table 2. There are 4 main modes of data collection in this study: (1) participant-completed ROM data collected via Smart Track (Multimedia Appendix 1), (2) broader app-generated data analytics summarizing interactions with Smart Track, (3) quantitative baseline and follow-up assessments with study participants, and (4) qualitative interviews with study participants and group facilitators.

Baseline and follow-up assessments will primarily be conducted over the telephone by a member of the research team (a trained clinical psychologist). To promote follow-up, appointments will be scheduled at a time convenient to the participants, with options to accommodate participants’ preferences for video link, face-to-face, and/or self-report (where feasible). Telephone, text, letter, and/or facilitator prompting will be utilized (as needed) to remind the participants of upcoming and/or missed appointments.

**Table 2:** Schedule of data collection.

Data collection method/instrument			Baseline		Daily		Weekly		2-week follow-up		2-month follow-up	
**SMART^a^ Recovery participants**												
	**Smart Track app**											
		Data analytics				✓						
		ROM items^b^						✓				
	**Telephone interview**											
	Demographics			✓								✓
	**Network of Alcohol and other Drug Agencies Client Outcome Management System**											
		Severity of Dependence Scale		✓								✓
		Drug and alcohol use		✓								✓
		Kessler 10+ scale		✓								✓
		The World Health Organization Quality of Life–8		✓								✓
		New South Wales minimum data set items (living arrangements and income)		✓								✓
		BTOM-C^c^ items on arrests		✓								✓
		BTOM-C items on risky drug using practices		✓								✓
	Substance Use Recovery Evaluator			✓								✓
	Client Services Receipt Inventory			✓								✓
	Mobile App Rating Scale–user version									✓		
	Digital Working Alliance Inventory									✓		✓
	Qualitative interview (n=20)											✓
**SMART Recovery facilitators**												
	Demographics											✓
	Mobile App Rating Scale–user version											✓
	Qualitative interview (n=10)											✓

^a^SMART: Self-Management and Recovery Training.

^b^See Multimedia Appendix 2 for a detailed description of routine outcome monitoring items as a function of assessment domain and frequency of administration.

^c^BTOM-C: Brief Treatment Outcome Measure-Concise.

### Data Handling and Storage

Initially, the ROM data entered by participants into Smart Track will be stored locally on the participants’ phones. When the participants connect to the internet, ROM data will be transmitted to a secure mobile and web app development platform (managed by SRAU), before transmitting to a secure server hosted by the University of Wollongong.

The participants’ responses to the baseline and follow-up research assessment instruments will be entered at the time of interview directly into REDCap, a secure web application for building and managing online surveys and databases. Further information about data management, monitoring, and dissemination is provided in Multimedia Appendix 3.

### Key Measures and Assessment Instruments

#### Primary Objectives: Feasibility and Acceptability

##### Mobile App Data Analytics

Mobile app data analytics will be captured daily throughout the study period using a mobile and web app development platform. Mobile app data analytics provide insight into how and when participants interact with an app (including participants’ interactions with the on-site tablets provided by the research team). In this study, analytics will be used to inform both feasibility (primary objective) and to describe usage patterns (secondary objective).

##### Qualitative Feedback

A qualitative evaluation (described under *Nested Qualitative Evaluation*) will be conducted 2 months postbaseline to collect detailed feedback from study participants and facilitators regarding their experience of and satisfaction with Smart Track.

##### App Quality Assessment

The Mobile App Rating Scale (MARS) [52] is designed to assess the quality of mHealth apps. The original version of this rating tool is designed to be completed by researchers, professionals, and/or clinicians [52]. The Mobile Application Rating Scale–User Version (uMARS [53]) is a simplified, end-user version. A total of 16 items are used to assess app quality across 4 domains (engagement, functionality, aesthetics, and information quality). Items are rated on a scale from 1 to 5 (1=*inadequate* and 5=*excellent*). Means are calculated for each quality domain and summed to produce an overall app quality mean score. Each instrument also contains 4 additional items to assess *subjective quality* and a further 6 items to assess the perceived impact of the app. Both the MARS [52] and uMARS [53] have excellent internal consistency and sound test-retest reliability.

The Digital Working Alliance Inventory (D-WAI) [54] is a brief, simple scale designed to assess therapeutic alliances within the context of app usage. It was recently developed to address the need for improved assessment of working alliances when evaluating the quality of digital health apps [54]. The D-WAI comprises 6 items and is derived from the short form of the Working Alliance Inventory [55], a commonly implemented and validated index of working alliances [56-58].

##### Cost Analysis

###### Health Services and Medication: Usage and Cost

An adapted version of the Client Service Receipt Inventory (CSRI)—*generic* UK mental health [59] will be used to assess health services and medication usage. The content of this inventory has been updated to reflect key sources of mental health expenditure in Australia [60]. These data will allow us to explore clinical and treatment characteristics that may be associated with app usage and will provide some insight into costing.

###### Time and Resource Utilization

Time and resource utilization will be captured in Microsoft Excel using a *cost capture template* [61-63]. This template will be used by the research team to maintain a record of cost data associated with the conduct of the study and the development and implementation of Smart Track. Only costs required to develop and implement the Smart Track app will be included in the cost analysis.

#### Secondary Objectives

##### Usage Patterns, Psychometric Properties, and Participant-Reported Outcomes

Secondary objectives will be informed by (1) participant-entered (and missing) data for Smart Track ROM items (detailed in Multimedia Appendix 2), (2) app-generated data analytics, and (3) the following data (eg sociodemographic characteristics; drug and alcohol use; health and social functioning; and recovery) collected by the research team at baseline and follow-up assessments.

##### Demographic Characteristics

Collection of sociodemographic characteristics (referral source, date of birth, gender, marital status, indigenous status, education/training, accommodation, and income) will be guided by items from the CSRI [59] and/or NSW minimum data set (MDS) for drug and alcohol treatment services [64].

The Network of Alcohol and other Drug Agencies (NADA) Client Outcome Management System (COMS) was developed by NADA to address the need for greater consistency in how outcomes are assessed across the drug and alcohol treatment sector [46]. We have chosen to use the COMS in this study to ensure that our data are directly comparable with the broader drug and alcohol treatment sectors. The COMS comprises a battery of items designed to assess 4 key domains: (1) drug and alcohol use, (2) psychological health, (3) health and social functioning, and (4) blood-borne virus (BBV) risk. The instruments used to assess each domain are outlined below. The COMS will be administered in full at baseline and at 2-month follow-up. A subset of items (Multimedia Appendix 2) will also be administered via Smart Track.

###### Client Outcome Management System: Drug and Alcohol Use

####### Severity of Dependence Scale

The Severity of Dependence Scale [65] is a 5-item screening measure of the psychological aspects of dependence that takes less than 1 min to complete. The items assess feelings of impaired control over drug taking, together with preoccupations and anxieties about drug taking. Participants will be asked to respond based on the substance that was causing them the greatest concern (1) over the preceding 2 months and (2) when they began attending SMART Recovery. The items are rated on a 4-point Likert scale, and total scores range from 0 to 15. Higher scores indicate a higher level of dependence. It is widely validated for use across a range of drug types, including heroin, cannabis, cocaine, amphetamine, and benzodiazepines [65-67].

####### Substance Use

Alcohol and tobacco use is measured by assessing both frequency (number of days) and quantity used during the preceding 4 weeks. Furthermore, 2 separate measurements for alcohol are included: number of days the person drank alcohol (and average number of drinks per day) and number of days of heavier drinking than usual (and average number of drinks on those days). For benzodiazepines and any illicit drugs, only the number of days of use is assessed.

###### Client Outcome Management System: Psychological Health

The Kessler 10 scale (K10) [68] is a widely used self-report measure of psychological distress. It comprises 10 questions that assess the level of nervousness, agitation, psychological fatigue, and depression in the past 4 weeks [68]. Each item is scored from 1 to 5, from *none of the time* to *all of the time*. Scores are then totaled, resulting in a K10 score between 10 and 50, with higher scores indicating greater distress [69]. The Kessler 10+ scale includes 4 additional questions to provide a context for interpretation (number of days where work, study, and/or management of daily activities were stopped and/or reduced because of these feelings; number of times professional help was sought; and perceived contribution of physical health problems to reported distress) [46]. The K10 has been successfully used in a range of populations, including a range of different Australian settings [69] and specifically with users of AOD in Australian settings [70].

###### Client Outcome Management System: Health and Social Functioning

####### World Health Organization Quality of Life Scale–8

The World Health Organization Quality of Life–8 [71] questions (also known as the EUROHIS QoL-8) is a very brief adaptation of the WHOQOL-100 and the WHOQOLBREF. Each item is scored from 1 (eg, not at all/very poor/very dissatisfied) to 5 (eg, completely/very good/very satisfied). Items are totaled (range 8-40), with higher scores reflecting greater perceived quality of life over the preceding 2 weeks. Domain scores can also be calculated for overall perception of the quality of life, overall perception of health, physical quality of life, psychological quality of life, satisfaction with social relationships, and satisfaction with the environment. It has been used and validated across a range of populations and settings [45,71], including AOD [72] and mental health [73].

####### New South Wales Minimum Data Set Items

To provide a differing and more objective assessment of the changes in the perceived quality of life [46], the COMS also includes two items on living arrangements (“Who do you live with?” and “Usual accommodation?”) and 1 item on income status (“What is your main source of income?”) taken from the NSW MDS [64]. Each item is answered by selecting one option from the response categories provided.

Two items on crime from the Brief Treatment Outcome Measure–Concise (BTOM-C) [47] are also included. These items were developed by the NSW Ministry of Health and assess the number of times the individual has been arrested in the last 2 months and how many arrests were for offenses committed in the preceding 2 months.

###### Client Outcome Management System: Blood-Borne Virus Risk

The BBV exposure risk-taking domain of the COMS comprises 4 items from the BTOM-C [47] on injecting drug use and overdose. These items are part of the validated BTOM measurement tool developed by NSW Health [47]. They are designed to measure changes and outcomes in relation to injecting and other risky drug use practices.

##### Client Perspectives of Recovery

The Substance Use Recovery Evaluator (SURE) [43] is designed to measure recovery from drug and alcohol dependence. It was developed in close consultation with people in recovery and comprises 21 items across 5 domains (drinking and drug use, self-care, relationships, material resources, and outlook on life). Items are summed to generate domain scores and an overall recovery score, with higher scores indicating greater progress toward recovery. Evidence supports face and content validity, acceptability, and usability for people in recovery [43]. We have selected the SURE as it is the first patient-reported outcome measure to provide a multidomain assessment of recovery, as defined by adults with experience of addiction [19]. Holistic assessment across a range of domains is consistent with both service user needs [74] and recovery-oriented service provision [19], thereby increasing the relevance of our findings to both service users and service providers. The SURE will be administered in full at baseline and at 2-month follow-up, with items also administered via Smart Track.

##### Nested Qualitative Evaluation

Qualitative interviews will be conducted 2 months postbaseline to explore the experience and opinions of participants with diverse engagement with Smart Track. Participants will be purposively sampled according to their baseline characteristics, pattern of Smart Track usage, and responses to the 2-month follow-up assessment. Specifically, we wish to explore the experience and opinions of 2 groups of SMART Recovery participants: (1) those who attended SMART groups regularly and completed the ROM regularly (n=10) and (2) those who attended SMART groups regularly and did not complete the ROM regularly (n=10). A qualitative researcher independent from the research team will use a topic guide to ask additional open-ended questions to the selection of participants (n=20) until the nominated sample size in both groups is reached. The research team will monitor recruitment to ensure that there is an adequate distribution of gender, main behavior of concern, geographical location, and group setting. Two corresponding groups of SMART Recovery facilitators (n=5 for each group) will also be recruited, namely (1) one group with members who regularly used Smart Track and (2) another group with members who did not regularly use Smart Track.

All interviews will be audio-recorded. A professional transcriber working under a confidentiality agreement will transcribe the recordings. The transcripts will be checked against the recordings for accuracy and deidentified (by removal of identifying information).

### Study Outcomes

Primary and secondary endpoints are presented in Tables 3 and 4, respectively.

**Table 3:** Primary endpoints.

Primary objective	Primary endpoint
To explore the feasibility of using Smart Track as part of SMART^a^ Recovery groups for the purposes of ROM^b^ and tailored feedback	Proportion of eligible participants who consent to the study Proportion of missing data for each of the ROM items/instruments at each week of administration, across the 2-month period of Smart Track usage Costs associated with developing Smart Track and maintaining the app until the completion of data collection Participant engagement with Smart Track, as indexed by data analytics captured daily across the data collection period
To explore the acceptability of using Smart Track as part of SMART Recovery groups for the purposes of ROM and tailored feedback	Detailed qualitative feedback from SMART Recovery group members and facilitators to explore their experience of and satisfaction with Smart Track (2-month follow-up) Quality ratings as assessed by participant and facilitator ratings of the user version of the Mobile App Rating Scale (2-week follow-up) and Mobile App Rating Scale (2-month follow-up), respectively Digital therapeutic alliance ratings as assessed by participant ratings of the Digital Working Alliance Inventory (2-week and 2-month follow-up)

^a^SMART: Self-Management and Recovery Training.

^b^ROM: routine outcome monitoring.

**Table 4:** Secondary endpoints.

Secondary objective	Secondary endpoints
To explore how participants engage with the app and describe usage patterns	Demographic, clinical, and treatment factors (as measured by Client Service Receipt Inventory, COMS^a^, and SURE^b^ at baseline and 2-month follow-up) associated with (in)completion of Smart Track ROM^c^ items Data analytics captured daily across the data collection period
To provide preliminary evidence for the psychometric properties of the ROM items administered by Smart Track	Internal reliability and convergent and divergent validity of COMS and SURE items administered by Smart Track (relative to the complete versions administered at baseline and 2-month follow-up)
To provide preliminary evidence for participant-reported outcomes in behaviors of concern, recovery, and mental health	Participant-reported progress across the 2-month period of app usage in the following: Addictive behaviors (COMS [41] and item adapted from the Screener for Substance and Behavioral Addictions [42]) Addiction recovery (SURE [43]) Mental health (Kessler [44,68])

^a^COMS: Client Outcome Management System.

^b^SURE: Substance Use Recovery Evaluator.

^c^ROM: routine outcome monitoring.

### Participant Reimbursement

Consistent with the Australian guidelines for acknowledging the time and value of consumer participation [75], participants will be offered modest reimbursement for any time, travel, and inconvenience associated with participation in the study assessments (supermarket voucher to the value of AUD $19.60 for baseline and 2-month follow-up assessments; AUD $1=US $0.65).

### Statistical Analysis

#### Primary Objectives

Given the primary objectives of exploring feasibility and acceptability, outcome data will primarily utilize descriptive statistics (eg, summarizing the recruitment rate, proportion of missing data, data analytics, MARS and uMARS quality ratings, and D-WAI alliance ratings). Descriptive statistics will be supplemented by the following cost and qualitative analyses.

##### Cost Analysis

A cost analysis will be conducted with the assistance of the health economics unit at the Hunter Medical Research Institute, Australia. The analysis will adopt a health provider perspective; it will measure and report the cost associated with developing and maintaining Smart Track. This is policy-relevant information as it estimates the resources required to translate the model of care to another location. Cost modeling will be conducted to report the direct costs of the additional resources required to develop and maintain Smart Track. The perspective adopted will be limited in the base case analysis to that of the health provider. Costs and resource use will be prospectively collected for the duration of the feasibility study and will be valued using a combination of hospital data from NSW Health, Medicare Benefits Schedule tariffs, and market rates. Downstream cost savings associated with hospitalization will also be explored.

##### Qualitative Evaluation

Qualitative data will be examined to inform the acceptability of Smart Track by exploring the participants’ and facilitators’ experiences of the perceived usefulness of ROM and any reason(s) for nonadherence. The analysis will proceed in 2 ways. First, we will identify key concepts and experiences to inform future development and/or refinement of Smart Track content, features, and/or procedures. Second, if there is sufficient data, an inductive approach [76] will be used to shed light on the ways in which individuals understand themselves and their actions (such as in the SMART Recovery group). The methodology also allows for individual beliefs and experiences to be positioned within broader social, service, and policy contexts, including factors such as drug treatment policy and service availability and social attitudes toward drugs and people who use them [77].

#### Secondary Objectives

##### App Engagement and Usage Patterns

A detailed exploration of the relationship between app usage (as indexed by frequency of ROM completion, number of missing items, and time to disengagement) and participant characteristics (demographic, clinical, and treatment variables) will be explored using linear regression. Furthermore, we intend to explore the ROM data graphically to see (1) whether it is likely to characterize particular patterns of usage and (2) whether these patterns appear to be influenced by various participant characteristics. Potential patterns that emerge during this exploratory phase will be followed up using latent trajectory analysis. This will clarify whether app use increases, decreases, or has some other pattern over time. Descriptive statistics will also be used to summarize app-generated data analytics.

##### Preliminary Psychometrics of Smart Track ROM Items

Preliminary psychometrics for Smart Track items will be explored via sensitivity to change, internal consistency, test-retest reliability, convergent validity, and exploratory factor analysis. Internal consistency of Smart Track ROM items will be evaluated using Cronbach alpha coefficient. Pearson correlation analysis will be used to examine the test-retest reliability of ROM scores for the first and second completion of each item set. Convergent validity will be examined using Pearson correlation analysis to explore the associations of initial ROM scores with the standardized measures (COMS and SURE) administered at baseline. Sensitivity to change will be examined via effect sizes, reliable change index (RCI), and growth curve modeling. As appropriate, internal consistency, test-retest reliability, concurrent validity, and RCI will be further examined as a function of age group, gender, and primary behavior of concern.

Effect sizes will be estimated for participants’ average ROM change scores between the first and last sessions. To explore the minimum reliable amount of change in scores (while accounting for measurement error [78]), we will calculate RCI between participants’ first and last ROM scores. The Jacobson and Traux criteria [78] will be applied to describe the proportion of participants who improved, did not change, or deteriorated. RCIs will also be calculated for the standardized measures administered at baseline and follow-up to allow comparisons with the ROM items. Growth curve modeling will be used to estimate the average rates of change in ROM scores across the 2-month period of ROM usage.

#### Method of Dealing With Missing Data

Descriptive analyses will use all available data. Inferential analyses (eg, linear regression and growth curve modeling) will use multiple imputation (with chained regression equations) as the primary method of dealing with missing data. The number of imputed data sets will depend on the fraction of missing data, but the stability of results will be assessed over a range of imputation numbers.

#### Participant Outcomes

Participants’ responses to the COMS and SURE (as captured via interview and ROM items across the 2-month data collection period) will be summarized using descriptive statistics.

#### Power

We aim to recruit participants from 13 sites that conduct a combined total of 25 groups per week. Assuming an average of 6 eligible participants per group per week and a target sample size of 100 participants, we anticipate recruitment to take between 4 and 6 weeks (for an estimated recruitment rate between 11% and 17%). A sample of this size will enable the estimation of the recruitment rate and 95% CI, with a margin of error of no more than 7%.

## Results

At the time of submission, 13 sites (25 groups per week) had agreed to be involved. Funding was awarded on August 14, 2017 and ethics approval was granted on April 26, 2018 (HREC/18/WGONG/34; 2018/099). Enrollment is due to commence in July 2019. Data collection is due to be finalized in October 2019.

## Discussion

### Principal Findings

Integrating ROM and tailored feedback into SMART Recovery groups is an important step toward improving client care [5,6,79]. Given the dearth of published research specifically examining the effectiveness of SMART Recovery [27], ROM provides the opportunity to establish an evidence base for SMART Recovery. Improved engagement with ROM and feedback requires innovative solutions [9]. To overcome the current limitations [9,18,80], in this project, we have harnessed technology to develop a fee-free mHealth app that provides interactive, client-centered, multidimensional progress monitoring. Written and visual feedback is generated automatically and is available almost instantly. Consistent with the need to improve the quality of mHealth solutions [81], Smart Track is grounded in theory, informed by an in-depth understanding of the needs and opinions of SMART Recovery participants and facilitators, and will undergo methodologically rigorous evaluation.

### Conclusions

To the best of our knowledge, this study will be the first to use ROM and tailored feedback within a mutual support group setting. Our study design will provide an opportunity to identify the acceptance of a novel mHealth ROM and feedback app within this setting and provide detailed information on the factors that help to promote or hinder the use of ROM within this context. The study will provide important contextual information to inform the development of future intervention studies focused on the effectiveness of adding ROM plus feedback to SMART Recovery. Further, should Smart Track prove feasible and acceptable, this project offers a new tool that service providers, policy makers and researchers could one day use to understand the impact of SMART Recovery.

## Appendix

Multimedia Appendix 1 Study reporting and registration.

Multimedia Appendix 2 Content and administration of routine outcome monitoring items.

Multimedia Appendix 3 Data management, monitoring and dissemination.

Multimedia Appendix 4 Roles and responsibilities.

Multimedia Appendix 5 Peer Review Summary by NSW Health.

## Abbreviations

AOD: alcohol and other drug
BBV: blood-borne virus
BTOM-C: Brief Treatment Outcome Measure-Concise
COMS: Client Outcome Management System
CSRI: Client Service Receipt Inventory
D-WAI: Digital Working Alliance Inventory
HREC: Human Research Ethics Committee
ISLHD: Illawarra Shoalhaven Local Health District
K10: Kessler 10 scale
MARS: Mobile App Rating Scale
MDS: minimum data set
mHealth: mobile health
NADA: Network of Alcohol and other Drug Agencies
NSW: New South Wales
RCI: reliable change index
ROM: routine outcome monitoring
SMART: Self-management And Recovery Training
SURE: Substance Use Recovery Evaluator
uMARS: Mobile Application Rating Scale–User Version
