# Supplementary material for: Feasibility of a Mobile Health App for Routine Outcome Monitoring and Feedback in Mutual Support Groups Coordinated by SMART Recovery Australia: Protocol for a Pilot Study
Source: JMIR Res Protoc. 2020 Jul 9;9(7):e15113. doi: 10.2196/15113 (PMC7380906; doi:10.2196/15113)
Supplement: Multimedia Appendix 2 [file resprot_v9i7e15113_app2.docx]

**Multimedia Appendix 2**

**Content and Administration of ROM items**

**Table 1.** Smart Track routine outcome monitoring items and response categories as a function of outcome domain and assessment frequency.

| Domain | Frequency | Instrument | Item(s) | Response option(s) |
| --- | --- | --- | --- | --- |
| Goal setting | Weekly (weeks A and B) | Adapted from the Change Plan [37,38] | Your 7-day plan: add tasks for this week | One or more tasks are added via free text and the participant is prompted to set the time and date for completing each task and whether/when they wish to set a reminder |
| Values | Weekly (weeks A and B; per 7-day plan task set) | Adapted from the Valued Living Questionnaire [39] | This task is important to me for family, intimate relationships, marriage, parenting, friendship, work, education, learning, recreation, spirituality, citizenship, community involvement, physical self-care, psychological self-care, and other | One or more domains are selected |
| Self-efficacy | Weekly (weeks A and B) | Confidence Ruler [37,38,40] | How confident do you feel about achieving this plan? | Sliding scale from 0-10 |
| Goal attainment | Weekly (weeks A and B) | Adapted from the Client Rating of Homework Performance [41] | Your 7-day plan: task X | For each task, progress is indexed by selecting “Done,” “Some,” or “Not yet started” |
| Group attendance | Weekly (weeks A and B) | —Generated by the Research Team | Not counting today, how many SMART Recovery groups have you gone to in the past 7 days? | 0-7 |
| **Frequency of addictive behavior** | | | | |

| Alcohol and other Drug Use | Weekly (weeks A and B) | Adapted from the COMS^a^ Questionnaire: Drug and Alcohol Use Scale [42]^b^ | Which of the following (if any) did you use this week? alcohol, cannabis, amphetamines, benzodiazepines, heroin, other opioid-based drug, cigarettes, other (please specify), or none | One or more response options are selected |
| --- | --- | --- | --- | --- |
|  | Weekly (weeks A and B) | Adapted from the COMS Questionnaire: Drug and Alcohol Use Scale [42]^b^ | How many days did you use each of the following? | Sliding scale (0-7) for each of the substances selected at item 6 |

| Other addictive behaviors | Weekly (weeks A and B) | Adapted from the Screener for Substance and Behavioral Addictions [43] | Think about the statement “I did it too much.” In the past 7 days, how often did this apply to gambling, shopping, sex, pornography, internet, food, video gaming, and other? | Sliding scale (0-7) for each of the behaviors listed |
| --- | --- | --- | --- | --- |
| Quantity of alcohol use^c^ | Weekly (weeks A and B) | Adapted from the COMS Questionnaire: Drug and Alcohol Use Scale [42]^b^ | On average, how many standard drinks did you have on those days when you were drinking? | A number from 0-100 is selected |
| Quantity of cigarette use^d^ | Weekly (weeks A and B) | Adapted from the COMS Questionnaire: Drug and Alcohol Use Scale [42]^b^ | How many cigarettes/cigars/pipes did you have on a typical day when you did use tobacco? _____ cigarettes/cigars/pipes | A number from 0-100 is selected |
| Alcohol and drug use: impact | Fortnightly (week A) | SURE^e^: Drinking and Drug Use Subscale [44] | Items 4-6 (eg, I have coped with problems without misusing drugs or alcohol) | “All of the time,” “Most of the time,” “A fair amount of the time,” “A little of the time,” or “None of the time” |
| Self-care | Fortnightly (week A) | SURE: Self-Care Subscale [44] | Entire subscale (items 7-11; eg, I have been taking care of my physical health) | e  “All of the time,” “Most of the time,” “A fair amount of the time,” “A little of the time,” or “None of the time” |
| Social support | Fortnightly (week A) | SURE: Relationships Subscale [44] | Entire subscale (items 12-15; eg, I have been getting on well with people | “All of the time,” “Most of the time,” “A fair amount of the time,” “A little of the time,” or “None of the time” |
| Resources | Fortnightly (week A) | SURE: Material Resources Subscale [44] | Entire subscale (items 16-18; eg, I have had stable housing) | “All of the time,” “Most of the time,” “A fair amount of the time,” “A little of the time,” or “None of the time” |
| Optimism | Fortnightly (week A) | SURE: Outlook on Life Subscale [44] | Entire subscale (items 19-21; eg, I have felt positive) | “All of the time,” “Most of the time,” “A fair amount of the time,” “A little of the time,” or “None of the time” |
| Mental health | Fortnightly (week B) | Kessler–6-item questionnaire [45] | Entire questionnaire | “None of the time,” “A little of the time,” “Some of the time,” “Most of the time,” or “All of the time” |
| Quality of life | Fortnightly (week B) | The World Health Organization Quality of Life–8 (EUROHIS-QOL 8-item index) [46] | Item 1: How would you rate your quality of life? | “Very Good,” “Good,” “Neither poor nor good,” “Poor,” or “Very Poor” |
| **Urges** | | | | |

| When | As needed | Adapted from The Urge Log [37,38] | — | Date and time are automatically captured when the participant clicks on the “Urge” button |
| --- | --- | --- | --- | --- |
| Intensity | As needed | Adapted from The Urge Log [37,38] | How intense is your urge? | “Low,” “Medium,” or “Strong” |
| Where | As needed | Adapted from The Urge Log [37,38] | What was going on? (eg, think about where you were, who was there, what was around you, and what you were doing) | Open text |
| Emotional Precursors | As needed | Adapted from The Urge Log [37,38] | How were you feeling? tired, stressed, hungry, bored, upset, lonely, relaxed, happy, excited, angry, worried, frustrated, energized, ashamed, or other | Select one or more of the experiences listed |
| Management | As needed | Adapted from The Urge Log [37,38] | Were you happy with how you handled it? | Yes/no |
| Strategies applied | As needed | Adapted from The Urge Log [37,38] | If yes, what worked for you this time? | Free text |
| Planned strategies | As needed | Adapted from The Urge Log [37,38] | If no, what would you like to do differently next time? | Free text |

^a^COMS: Client Outcome Management System [47].

^b^This subscale of the Client Outcome Management System is derived from the Brief Treatment Outcome Measure [48].

^c^Only if the participant endorses *alcohol* use over the preceding 1 week (item 6).

^d^Only if the participant endorses *cigarette* use over the preceding 1 week (item 6).

^e^SURE: Substance Use Recovery Evaluator [44].
