# Supplementary material for: Feasibility of a Mobile Health App for Routine Outcome Monitoring and Feedback in Mutual Support Groups Coordinated by SMART Recovery Australia: Protocol for a Pilot Study
Source: JMIR Res Protoc. 2020 Jul 9;9(7):e15113. doi: 10.2196/15113 (PMC7380906; doi:10.2196/15113)
Supplement: Multimedia Appendix 3 [file resprot_v9i7e15113_app3.docx]

Multimedia Appendix 3

Data Management, Monitoring and Dissemination

Each participant will be assigned a unique alphanumeric code. This code will be used to label their data for collection, storage, transmission and analysis. The encrypted file containing the link between participant details and each unique alphanumeric code will be password protected, stored separately to study data and accessible only to key research personnel. Statistical analyses will be conducted on deidentified data only. Data will be used, stored and destroyed as per HREC approved methods and UOW guidelines.

*Access to Data*

Data management and sharing will be overseen by trial co-ordinator A.K. Beck in collaboration with Chief Investigator P.J Kelly. All named investigators will have access to the cleaned data set. Data sharing will be managed using REDCap. The University of Wollongong (UOW) recommends use of the UOW repository within six months from publication of research findings.

Monitoring

An independent data monitoring committee will not be convened. The current study is a one-arm pilot evaluation of a non-invasive process for monitoring treatment outcomes. Smart Track has been developed in close consultation with participants to reflect evidence based principles and strategies, will be utilised within the context of ‘treatment as usual’ and does not involve experimental administration of medicine or experimental therapeutic devices. No interim analyses are planned.

*Potential Harms and Unintended Consequences*

*Dealing with Risk*

Study procedures are benign and closely mirror elements of usual practice (questionnaires, interviews, discussions etc). Accordingly, this is a low-risk, low-burden study where the only potential risks include minor inconvenience and a very small risk of distress. Procedures have been implemented to minimise the occurrence of either (e.g. selecting brief assessment instruments, accommodating preferred mode of interview administration where possible and acknowledging participant contribution via modest reimbursement).

*Assessment, Documenting and Reporting of Adverse Events*

Should a participant report concerns about study procedures (spontaneously during study participation, or upon enquiry at the follow-up interview), including any actual or perceived privacy breaches this information will be documented and as needed, advice from the responsible HREC will be sought.

*Ancillary and Post-Trial Care*

In the unlikely event of harm (directly attributable to study procedures), participants enrolled into the study will be covered as per the conditions set out in The University of Wollongong and SMART Recovery Medical Malpractice & Professional Indemnity and Public Liability insurance policies.

*Auditing*

Progress reports will be submitted to the funding body and HREC as required. The steering committee will meet approximately every six months across the duration of the study (at a minimum).

*Bug fixes, Downtimes and Content Changes*

The research team will maintain a log of any major i) bug fixes, ii) changes in the functionality or content of Smart Track and any iii) system failures and downtimes across the duration of the pilot study.

Dissemination Policy

The major outcome from the proposed study will be Smart Track. This will be a world first mHealth ROM and Feedback App developed specifically for use as part of mutual support groups. We intend to develop a simple protocol to help participants and SMART Recovery facilitators to use the tool. Training resources will also be developed by the team and will help to inform our dissemination activities (e.g. workshops).

Study data will also be used in a range of dissemination activities. The most effective way to disseminate information across the broader NSW NGO sector is through presentations at the NADA conference or through NADA sponsored training forums. The team will also submit abstracts for APSAD, the leading industry conference. It is our intention to develop and submit academic papers for publication throughout the study period. Research articles will be disseminated across the sector via the NADA Practice Leadership Group and the ACI Drug and Alcohol Network (AI P.J. Kelly is a member of both committees). All prepublication versions of articles will be made available through the University of Wollongong research depository. A lay summary of study findings will also be produced and distributed directly to participants who requested this at the time of consent. A lay summary will also be distributed to SMART Recovery facilitators.
