# Supplementary material for: Feasibility of a Mobile Health App for Routine Outcome Monitoring and Feedback in Mutual Support Groups Coordinated by SMART Recovery Australia: Protocol for a Pilot Study
Source: JMIR Res Protoc. 2020 Jul 9;9(7):e15113. doi: 10.2196/15113 (PMC7380906; doi:10.2196/15113)
Supplement: Multimedia Appendix 4 [file resprot_v9i7e15113_app4.docx]

Multimedia Appendix 4

Roles and Responsibilities

*Protocol Contributors*

Authorship follows ICMJE recommendations [82]. All authors made substantial contributions to study conception, design, methods and/ or the content and features of the routine outcome monitoring and feedback system under investigation. P.J. Kelly is chief investigator and contributed his expertise on the development, implementation and evaluation of evidence based approaches within substance abuse treatment settings. A.K. Beck is trial coordinator and led the development of the study protocol in collaboration with all investigators listed. A.L. Baker contributed her expertise on novel interventions for that target multiple health behaviour change including co-existing mental health and substance use problems. F.P. Deane contributed his expertise on factors that improve psychosocial treatments for mental health and/ or substance related difficulties, including the assessment and implementation of recovery-oriented mental health care. L. Hides contributed her expertise and experience from developing and evaluating web and mobile phone based programmes for the treatment of primary and comorbid substance use disorders. V. Manning contributed her expertise in trialling novel interventions to improve outcomes for substance dependent clients and those with co-occurring disorders, including the role of peer support. A. Shakeshaft contributed his expertise on embedding the evaluation of interventions into the delivery of routine clinical health services and cost effectiveness evaluations of tailored feedback. B. Larance contributed her expertise in the design and conduct of epidemiological and clinical studies designed to improve the health and wellbeing of people who misuse substances. J. Neale contributed her expertise in quantitative and qualitative methods for developing and evaluating patient reported outcome measures for adults with experience of addiction. . J. Kelly contributed his expertise in addiction treatment, the recovery process and mechanisms of change. C. Oldmeadow will lead the quantitative statistical analysis and has provided expert advice on study design. A. Searles will lead the economic evaluation and has provided expert advice on study design to inform the economic analysis. Treloar provided local qualitative expertise, including overseeing the development and implementation of the qualitative methodology. R.M. Gray contributed her qualitative expertise and led the collection and evaluation of qualitative data. A. Argent contributed her expertise on the conduct and content of SMART Recovery groups and strong track record in translating research evidence into practice. R. McGlaughlin contributed his expertise on the conduct and content of SMART Recovery groups and strengths-based and harm minimisation practices and tools more broadly. All authors also offered critical revisions to the manuscript for important intellectual content, have approved the final version of this manuscript and agree to be accountable for all aspects of the work in ensuring that questions related to the accuracy or integrity of any part of the work are appropriately investigated and resolved.

*Advisory Committees*

The expert advisory committee is comprised of study investigators P.J. Kelly, A.L. Baker, F.P. Deane, A. Shakeshaft, L. Hides, V. Manning, A.K. Beck, B. Larance, A. Argent, R. McGlaughlin, and the Head of Digital and Brand at SMART Recovery (Michael Bellamy).

The steering committee is comprised of investigators P.J. Kelly, A.K. Beck and R. McGlaughlin and representatives from Lives Lived Well (Dr Julaine Allan), The Network of Alcohol and Other Drugs Agencies (Dr Suzie Hudson), Griffith Aboriginal Medical Service (Sid Barone), The Centre for Addiction Medicine (Mr Angelo Barbaro) and Waverley Drug and Alcohol Centre (Eithne Cornish).
