# Supplementary material for: Feasibility of a Mobile Health App for Routine Outcome Monitoring and Feedback in Mutual Support Groups Coordinated by SMART Recovery Australia: Protocol for a Pilot Study
Source: JMIR Res Protoc. 2020 Jul 9;9(7):e15113. doi: 10.2196/15113 (PMC7380906; doi:10.2196/15113)
Supplement: Multimedia Appendix 5 [file resprot_v9i7e15113_app5.pdf]

H17/51861-7

rmcglaughlin@srau.org.au

Dear Mr McGlaughlin

Re: AOD Innovation Grants Scheme Round 1 outcomes

I am delighted to advise that your application to Round 1 of the AOD Innovation Grants Scheme for the project 'Routine outcome monitoring (ROM) plus feedback in SMART Recovery Australia: a feasibility study examining SMART ROM' has been successful.

Your proposal was one of five full applications received in the current round. All full applications were assessed through a competitive process by the AOD Innovation Grants Scheme Independent Selection Panel, with advice from other relevant experts where required.

The Selection Panel has requested some minor adjustments to your proposal, outlined in the attached document. These requests are made with the intention of ensuring the best possible outcome for your research project. Please review the feedback, incorporate the changes in track on your original full application and return a pdf and word version to aodgrants@moh.health.nsw.gov.au as soon as possible.

A Selection Panel member will be required to review and endorse the revised application. Once endorsed, Genevieve Whitlam, Senior Program Manager, will contact you regarding the final funding amount and your Funding Agreement. The Ministry will need to be notified of progress of the research project and financial acquittal of grant funds, including notification of any unspent funds. Reporting requirements for the grant are outlined in the Funding Agreement. Please also notify the Ministry if you receive other sources of funding for this research proposal.

I urge you to start any processes for recruitment of project staff, and ethics and governance approval as soon as you are advised that your revised application has been endorsed by a Selection Panel member. Given the relatively short timeframes of the grant period, starting these processes as soon as possible will have a significant impact on project timelines.

NSW Health will be announcing the successful applicants shortly. Until this announcement please do not initiate local media in regard to the award of this fund.

For any queries or feedback please email aodgrants@moh.health.nsw.gov.au.

Congratulations on the success of your full application. Thank you for your commitment to research that will contribute to the health and well-being of people in NSW. I look forward to updates on the progress of your research project.

Yours sincerely

Dr Kerry Chant PSM
Chief Health Officer and Deputy Secretary
Population and Public Health

14/8/17

NSW Ministry of Health
ABN 92 697 899 630

73 Miller St North Sydney NSW 2060
Locked Mail Bag 961 North Sydney NSW 2059
Tel. (02) 9391 9000 Fax. (02) 9391 9101
Website. www.health.nsw.gov.au

AOD Innovation Grants Scheme Full Application: Selection panel comments

Application Number:	207
Chief Investigator:	Ryan McGlaughlin
Host Organisation:	SMART Recovery Australia
Project Title:	Routine outcome monitoring (ROM) plus feedback in SMART Recovery Australia: a feasibility study examining SMART ROM
Budget requested:	$358,913

The following feedback from the AOD Innovation Grants Scheme Independent Selection Panel should be incorporated into the final research project proposal before it commences. Milestone reports for the funds at the end of year one and two will need to demonstrate that these comments have been addressed.

- Thank you for incorporating the data linkage component as requested by the Ministry. However, as your proposal has been re-designed as a feasibility study, the Selection Panel requests that you remove the data linkage component, for this particular proposal. It might be an approach to consider as part of any future efficacy study.
- The Selection Panel encourages you to consider increasing your sample size for the qualitative assessment to ensure a representative and sufficient sample.
- If the changes requested above impact your proposed budget, please revise accordingly.
